# Feasibility of Monitoring Patients Who Have Cancer With a Smart T-shirt: Protocol for the OncoSmartShirt Study

**DOI:** 10.2196/37626

**Published:** 2022-10-03

**Authors:** Emma Balch Steen-Olsen, Helle Pappot, Allan Green, Henning Langberg, Cecilie Holländer-Mieritz

**Affiliations:** 1 Department of Oncology Rigshospitalet University Hospital of Copenhagen Copenhagen Ø Denmark; 2 Knowledge Center of Telemedicine Region Hovedstaden Hillerød Denmark; 3 Department of Innovation Rigshospitalet University Hospital of Copenhagen Copenhagen Ø Denmark

**Keywords:** biometric sensor technology, cancer, home monitoring, patient-generated health data, sensor, smart t-shirt, remote monitoring, adolescent, protocol, patient, youth, health care professional, cancer treatment

## Abstract

**Background:**

Studies have shown that there may be dissimilar perceptions on symptoms or side effects between patients with cancer and health care professionals. This may lead to symptomatic patients notifying the clinic irregularly or not telling the clinic at all. Wearables could help identify symptoms earlier. Patients with low socioeconomic status and less self-awareness of their health may benefit from this. A new design of wearables is a smart t-shirt that, with embedded sensors, provides measurement flows such as electrocardiogram, thoracic and abdominal respiration, and temperature.

**Objective:**

This study evaluates the feasibility of using a smart t-shirt for home monitoring of biometric sensor data in adolescent and young adult and elderly patients during cancer treatment.

**Methods:**

The OncoSmartShirt study is an explorative study investigating the feasibility of using the Chronolife smart t-shirt during cancer treatment. This smart t-shirt is designed with multiple fully embedded sensors and electrodes that engender 6 different measurement flows continuously. A total of 20 Danish patients with cancer ≥18 years old in antineoplastic treatment at Department of Oncology Rigshospitalet Denmark will be recruited from all cancer wards, whether patients are in curative or palliative care. Of these 20 patients, 10 (50%) will be <39 years old, defined as adolescent and young adult, and 10 (50%) will be patients >65 years old, defined as elderly. Consenting patients will be asked to wear a smart t-shirt daily for 2 weeks during their treatment course.

**Results:**

The primary outcome is to determine if it is feasible to wear a smart t-shirt throughout the day (preferably 8 hours per day) for 2 weeks. Inclusion of patients started in March 2022.

**Conclusions:**

The study will assess the feasibility of using the Chronolife smart t-shirt for home monitoring of vital parameters in patients with cancer during their treatment and bring new insights into how wearables and biometric data can be used as part of symptom or side-effect recognition in patients with cancer during treatment, with the aim to increase patients’ quality of life.

**Trial Registration:**

ClinicalTrials.gov NCT05235594; https://beta.clinicaltrials.gov/study/NCT05235594

**International Registered Report Identifier (IRRID):**

PRR1-10.2196/37626

## Introduction

Collecting biometric sensor data by wearables is an example of real-time patient-generated health data that can provide vital and detailed objective information about patients. Although previous results from our research group have shown that the literature on wearables is very heterogeneous and lacks consensus [1], studies show that wearables may have the potential to improve quality of oncological treatment and increase patients’ quality of life [2-5].

During oncological treatment, most patients are primarily seen in outpatient clinics where the number of visits is determined by type of treatment and the expected side effects [6]. The symptoms and side effects, experienced by patients with cancer, depend on the type of cancer, the treatment modality, and the preexisting comorbidity [7-9]. Patients are informed to notify the clinic if they experience side effects or increased symptoms. In worst case scenarios, patients may need acute hospital treatment, while in other cases, side effects are more related to poorer treatment compliance and reduced quality of life [2].

A wearable is a noninvasive and wireless sensor device that can monitor and collect health parameters on various biometric data points such as skin temperature, respiration rate, heart rate, and physical activity. The device normally transmits the health data to an app (eg, on a smartphone), which registers the readings. The devices can be worn in different ways, depending on the design of the device (eg, a smartwatch around the wrist) [10,11].

Thus, wearables offer the opportunity of monitoring patients passively in their own environment while they are outside the hospital. This allows the patients to carry on with their daily life and thus minimize the burden from the decrease in their quality of life [12].

Studies have shown that there may be dissimilar assessments and perceptions on symptoms between patients and health care professionals [13,14], which is reflected by the fact that health care professionals often underestimate patients’ symptoms [15]. This may cause patients with symptoms or side effects to notify the clinic irregularly or not to notify the clinic at all, which could lead to unnecessary discomfort for patients and suboptimal treatment. In such cases, wearables could help identify symptoms or side effects earlier. In particular, patients with low socioeconomic status and less self-awareness of their health are assumed to benefit from using wearables [3].

A new design of a wearable is the smart t-shirt. A smart t-shirt is designed with sensors embedded into the fabric, which allows for the 24/7 monitoring of electrocardiogram (ECG), thoracic and abdominal respiration, and so on. Originally, smart t-shirts were designed to support athletes in their performance analysis and preventing injuries but with an ambition to support and improve health care as a wearable medical device [16]. Compared to other wearables, the smart t-shirt enables health professionals to monitor an increased number of health parameters on various biometric data points. Furthermore, data collection is predicted to be exact and completely comparable to conventional medical measuring devices [17,18]. Studies have shown that smart t-shirts can monitor 12-lead ECG acquisition with the same quality of standard Holter recording [17-19].

These new technologies allow health professionals to track patients’ health more extensively. At the same time, the new tools provide precise information without recall and reporting bias, which can lead to a better and more accurate cancer treatment [3,20-22]. However, knowledge in using wearables in an oncological setting is limited [12,20,23,24], and it has been highlighted that there is very little consensus and awareness of adherence to wearables. This is an essential part of being able to use and compare collected biometric sensor data [1], and it could be questioned whether oncological patients are able to adhere to the use of a smart t-shirt during their treatment.

In this paper, the study design of the OncoSmartShirt feasibility study is described. The purpose of this study is to evaluate the feasibility of using a smart t-shirt for remote monitoring of biometric sensor data in adolescent and young adult (AYA) and elderly patients during cancer treatment.

## Methods

### Study Design

The OncoSmartShirt study is an explorative study investigating the feasibility of using the Chronolife Smart t-shirt during cancer treatment. This smart t-shirt is designed with multiple sensors and electrodes fully embedded, which engender 6 different measurement flows continuously [25].

This trial is an investigator-driven partnership between Department of Oncology Rigshospitalet and Chronolife and is registered at ClinicalTrials.gov (NCT05235594). The study is conformed to the guidelines of General Data Protection Regulation and is registered at the Capital Region of Denmark (P-2021-357). The trial is approved by the local division for IT and Medico Technology in the Capital Region of Denmark and is a collaboration between Department of Oncology Rigshospitalet, Department of Innovation Rigshospitalet, and Telemedical Knowledge Center Capital Region of Denmark. Approval from the National Committee on Health Research Ethics is not required for this trial in the Danish context.

The acceptance and comfort of wearing the Chronolife smart t-shirt throughout the day (preferably 8 hours per day) for 2 weeks is investigated in all 20 enrolled patients. The intervention period will elapse at any time in the patient’s antineoplastic treatment course.

Secondly, qualitative telephone interviews will be carried out, and patients will be asked to fill in a questionnaire concerning their experience with wearing the shirt. Topics included in the interviews are, among others, the material and design of the smart t-shirt, feeling social stigma, and surveillance. The interview guide and patient questionnaire are available in Multimedia Appendices 1 and 2, respectively. The study will be performed in a public health care system, and the smart t-shirt will be given to the patients by the hospital. Only the patient included in the trial will be allowed to wear the t-shirt during the study period. Patients will be responsible for charging and washing the shirt and are required to return the shirt at study termination. No biometric data of vital parameters collected by the wearable will be published or monitored by health care professionals during the study. All other interventions, such as oncological care and treatment, will be kept to their normal routine. Participation in the study will not result in any payment or reward for the included patients.

### Patients and Recruitment

A total of 20 Danish patients ≥18 years old who have cancer and are in antineoplastic treatment will be recruited continuously. Of these 20 patients with cancer, 10 (50%) will be <39 years old, defined as AYA, and 10 (50%) >65 years old, defined as elderly, will be included. These 2 age groups were included, as we expected that these would differ the most from each other in terms of acceptance of the smart t-shirt. There will be no requirements regarding specific cancer diagnosis, and both patients in curative and palliative care will be included meaning that all types of patients with cancer can be included. These broad inclusion criteria are designed to make inclusion in the study as simple as possible. Therefore, recruitment of patients with cancer in the study will take place consecutively in all cancer departments at the Department of Oncology Rigshospitalet, Denmark. Patients will be eligible if they read and speak Danish and have the capacity to provide written informed consent to participate in the study. Patients can withdraw their consent at any time. Patients with serious cognitive deficits and who cannot reliably provide informed consent will be excluded. Inclusion in the study will have no interference with the planned oncological treatment.

### Hardware

The study device in the trial consists of four units; a washable smart t-shirt fitted with multiple sensors and electrodes from Chronolife, a companion smartphone or tablet app, an accredited secure data hosting server, and a web interface [25]. The Chronolife smart t-shirt is commercialized and “CE marked” for the consumer market. The smart t-shirt is designed for every-day use. The electrical sensors embedded in the shirt allows detection of 6 physiological parameters: ECG (beat per minute), thoracic and abdominal respiration (respiration per minute), thoracic impedance (kOhm), physical activity (steps), and skin temperature (°C) [25]. The sensors are powered by a nonremovable rechargeable battery. Additionally, the t-shirt is equipped with a memory card that stores data and a Bluetooth interface that transmits data. These are fully integrated into the t-shirt and have been sealed in water-resistant coatings.

### Software

**Figure 1:**
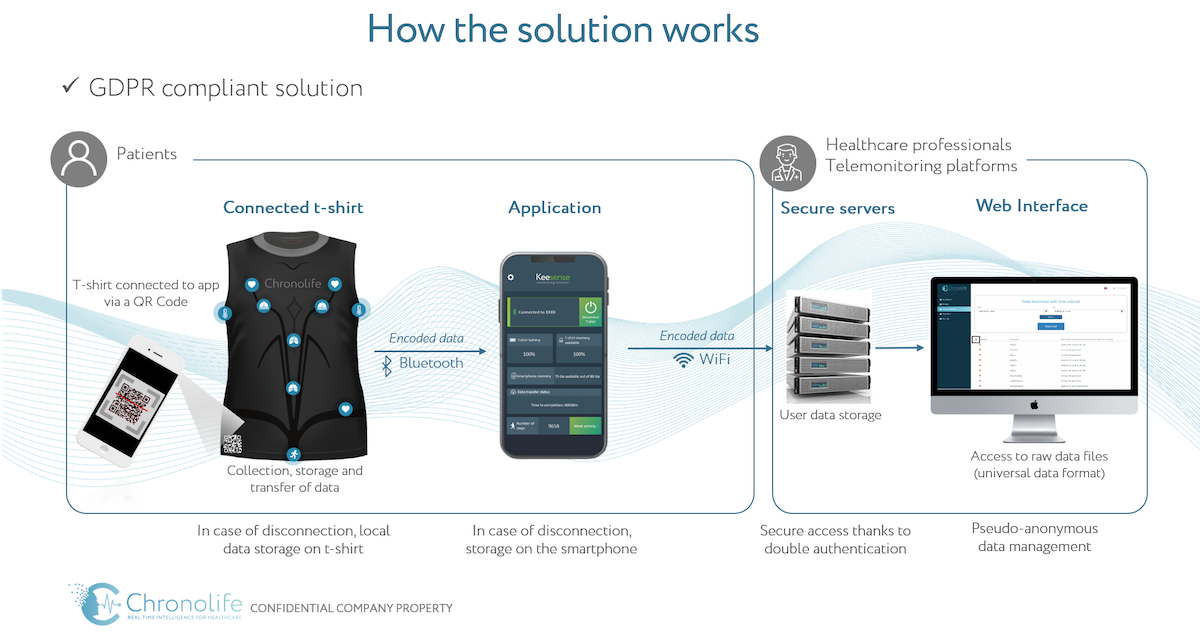
Data flow from data collected by the t-shirt to the smartphone app to the accredited data hosting server. Illustration made public with allowance from Chronolife. GDPR: General Data Protection Regulation.

The smart t-shirt connects to the smartphone app via a QR code located on the shirt. The health data collected by the sensors in the shirt are transmitted by Bluetooth Low Energy to the connected smartphone app designed for storage (Figure 1). Furthermore, the smartphone app provides further data transmission through 3G or 4G Wi-Fi to an accredited data-hosting server that will store data and provide data for a web interface for analysis and algorithm training [25]. Figure 1 illustrates the four components and the flow of data.

### Statistical Analysis

#### Endpoints

The primary endpoint is to assess the feasibility of using the Chronolife smart t-shirt based on completion rate, which in this trial is defined as the number of included patients using the smart t-shirt at least 8 hours per day during the 2 weeks study period.

Secondary endpoints are to assess technical feasibility in a Danish health care system, including data acquisition rate and data completeness. Qualitative interviews with the patients regarding the use of the smart t-shirt will be performed. Patients will be asked to fill in a questionnaire concerning their experience with wearing the t-shirt. As explorative endpoint changes in heart rate, skin temperature, physical activity, and respirations frequency will be presented descriptively.

Descriptive data will be collected and analyzed in the statistical software SPSS Statistics (IMB Corp).

#### Power

A power calculation is not required for this type of study because it is a feasibility study with no control group and no formal statistical hypothesis testing, and thus, the sample size is not driven by formal power calculations. This sample size corresponds to studies of the same nature in the literature [26-28].

### Ethical Considerations

The inclusion of patients will not begin until the Data Protection Agency has granted their relevant approval for the study. Patients will receive verbal and written information and must provide written informed consent. Moreover, they can withdraw at any time during the study. The Scientific Ethics Committees for the Capital Region of Copenhagen has been informed; however, approval is not required for this type of study according to Danish law.

## Results

Inclusion of patients started in March 2022. Data collection is expected to be completed in autumn 2022. Processing of data is expected to begin in autumn 2022 as well.

## Discussion

The study will assess the feasibility of using the Chronolife smart t-shirt for remote home monitoring of vital parameters in AYA and elderly patients during their treatment course. This study will bring new insights into how wearables and biometric data potentially can be used as a part of symptom recognition in patients with cancer during the treatment course in the quest of increasing their quality of life, given that the use of smart t-shirts is feasible. Data from this study can be used in designing future prospective studies using the smart t-shirt as intervention, along with the recommendation from the Clinical Trials Transformation Initiative on Developing Novel Endpoints Generated by Mobile Technologies for Use in Clinical Trials 2017. In future studies, it would be relevant to examine the relationship between biometric data collected from wearables and the perception of symptoms and side effects from patients and clinicians.

## Appendix

Multimedia Appendix 1 Interview guide.

Multimedia Appendix 2 Patient questionnaire.

## Abbreviations

AYA: adolescent and young adult
ECG: electrocardiogram

## Data Availability

Data from this study are not yet available. Data will not be publicly available due to institutional restrictions.
