# Supplementary material for: Feasibility of Monitoring Patients Who Have Cancer With a Smart T-shirt: Protocol for the OncoSmartShirt Study
Source: JMIR Res Protoc. 2022 Oct 3;11(10):e37626. doi: 10.2196/37626 (PMC9577710; doi:10.2196/37626)
Supplement: Multimedia Appendix 1 [file resprot_v11i10e37626_app1.pdf]

**Fra baggrundslitteraturen**	**Emne**	**Spørgsmål**
	Umiddelbare oplevelser af T-shirten	Hvordan har dine oplevelser været af T-shirten og af at gå med den?
Fordele:<br>- Selvmonitorering<br>- Adherence<br><br>*Ulemper:*<br>- Kan ikke være fysisk aktiv med den på<br>- Kun behagelig et par timer ad gangen	Fordele og ulemper	Hvilke fordele synes du, der var ved T-shirten?<br><br>Hvilke ulemper synes du, der var ved T-shirten?
Skal ikke være synligt, kradse, sidde tæt eller være varmt<br><br>Udseendes betyder noget, specielt for yngre målgrupper<br><br>- Kan ikke være fysisk aktiv med den på<br>- Kun behagelig et par timer ad gangen	Materiale og udseende	Hvordan var T-shirten at have på?<br><br>Hvordan vurderer du den udseende?<br><br>Hvordan vurderer du dens materiale?<br><br>Kunne du være fysisk aktiv i T-shirten, og hvordan var det? (Blev den tung, klæbrig, varm eller ildelugtende?)
Må ikke ligne patienttøj<br><br>Patientidentitet<br><br>Social stigma	Identitet<br>*Patientliggørelse*<br>*Social stigmatisering*	Hvad gjorde det ved dig at gå med T-shirten?<br><br>Blev T-shirten bemærket af andre? (Hvordan var det?)<br><br>Mindede T-shirten dig om, hvorfor du havde den på? (Var det en konstant påmindelse om kræften?)<br><br>Følte du dig ekstra patientliggjort, da du bar T-shirten?<br><br>Følte du dig som en anderledes person med T-shirten på? (fx som en prima-patient?)<br><br>Følte du dig på nogen måde stigmatiseret, da du gik med T-shirten?

- Adherence<br>- Holder selv øje med "progress" i aktivitet	Adfærdsændringer	Tænkte du over, at du havde den på, eller kunne du glemme den?<br><br>Ændrede du adfærd i løbet af de 2 uger, du gik med T-shirten?<br><br>Fx Trak du vejret anderledes?<br><br>Fx Var du mere eller mindre fysisk aktiv?<br><br>Holdt du øje med dine egne data? (Hvor ofte?)
Svært at abstrahere fra overvågningsaspektet og hvor data bliver gemt	Etik	Følte du dig overvåget?<br><br>Tænkte du over, hvor og hvordan dine data blev registreret og gemt?
	Egne tilføjelser	Er der noget, du gerne vil fortælle om, evt. som vi ikke har været inde på?

Baggrundslitteratur:

- Bergmann JHM, Chandaria V, McGregor AH. Wearable and implantable sensors: the patient's perspective. Sensors 2012;12:16695–709
- Papi E, Belsi A, McGregor AH. A knee monitoring device and the preferences of patients living with osteoarthritis: a qualitative study. BMJ Open. 2015 Sep 7;5(9):e007980.-
- Turhan G. An assessment towards the acceptance of wearable technology to consumers in Turkey: the application to smart bra and t-shirt products. The Journal of The Textile Institute. 2013, 104:4, 375-395, DOI: 10.1080/00405000.2012.736191
- Park S, Harden AJ, Nam J, Saiki D, Hall SS, Kandiah J. Attitudes and Acceptability of Smart Wear Technology: Qualitative Analysis from the Perspective of Caregivers [Internet]. Vol. 13, International Journal of Human Ecology. The Korean Home Economics Association; 2012. p. 87–100.
- Wagner, Basran, J., & Dal Bello-Haas, V. (2012). A review of monitoring technology for use with older adults. *Journal of Geriatric Physical Therapy (2001)*, *35*(1), 28–34. https://doi.org/10.1519/JPT.0b013e318224aa23
- J. Kubicek *et al.*, "Recent Trends, Construction, and Applications of Smart Textiles and Clothing for Monitoring of Health Activity: A Comprehensive Multidisciplinary Review," in *IEEE Reviews in Biomedical Engineering*, vol. 15, pp. 36-60, 2022, doi: 10.1109/RBME.2020.3043623.
