# Supplementary material for: Feasibility of Monitoring Patients Who Have Cancer With a Smart T-shirt: Protocol for the OncoSmartShirt Study
Source: JMIR Res Protoc. 2022 Oct 3;11(10):e37626. doi: 10.2196/37626 (PMC9577710; doi:10.2196/37626)
Supplement: Multimedia Appendix 2 [file resprot_v11i10e37626_app2.pdf]

Date questionnaire completed __ / __ / __

Perception and satisfaction evaluation questionnaire

Patient

OncoSmartShirt study: Continuous medical telemonitoring of cancer patients using the Chronolife^{TM} smart t-shirt.

Instructions

In this questionnaire, you will find questions on the use of the smart t-shirt as part of the evaluation of the OncoSmartShirt study.

Please answer the following questions or tick the answer that seems most appropriate to you in relation to your condition, your feelings, what you think.
For each of the following items, indicate the extent to which you totally disagree, tend to disagree, tend to agree or totally agree

Responses will be measured by a 5-point scale where 1 corresponds to "Totally disagree" and 5 to "Totally agree".

1	2	3	4	5
Strongly	disagree	Neutral	Slightly agree	Strongly agree
☐	☐	☐	☐	☐

There are no correct or incorrect answers. Thank you for answering all of the questions.

Date questionnaire completed __ / __ / __

General questions at the beginning of the evaluation

<table>
<tr><th>Elements</th><th>Questions</th></tr>
<tr><td rowspan="5">Pre-evaluation</td><td>1. Before starting the project, I had no fears regarding the medical device – the smart t-shirt and the phone.
<table><tr><td>1</td><td>2</td><td>3</td><td>4</td><td>5</td></tr><tr><td>☐</td><td>☐</td><td>☐</td><td>☐</td><td>☐</td></tr></table></td></tr>
<tr><td>2. I think using this smart t-shirt will help my doctors monitor my condition more closely.
<table><tr><td>1</td><td>2</td><td>3</td><td>4</td><td>5</td></tr><tr><td>☐</td><td>☐</td><td>☐</td><td>☐</td><td>☐</td></tr></table></td></tr>
<tr><td>3. I am afraid that using the smart t-shirt may affect my daily activities.
<table><tr><td>1</td><td>2</td><td>3</td><td>4</td><td>5</td></tr><tr><td>☐</td><td>☐</td><td>☐</td><td>☐</td><td>☐</td></tr></table></td></tr>
<tr><td>4. I think using the smart t-shirt can help me be more active.
<table><tr><td>1</td><td>2</td><td>3</td><td>4</td><td>5</td></tr><tr><td>☐</td><td>☐</td><td>☐</td><td>☐</td><td>☐</td></tr></table></td></tr>
<tr><td>5. I am concerned about a possible malfunction of the smart t-shirt.
<table><tr><td>1</td><td>2</td><td>3</td><td>4</td><td>5</td></tr><tr><td>☐</td><td>☐</td><td>☐</td><td>☐</td><td>☐</td></tr></table></td></tr>
<tr><td></td><td>6. After being shown and taught how to use the smart t-shirt, I was confident that I could then use it.
<table><tr><td>1</td><td>2</td><td>3</td><td>4</td><td>5</td></tr><tr><td>☐</td><td>☐</td><td>☐</td><td>☐</td><td>☐</td></tr></table></td></tr>
<tr><td colspan="2">Comments:</td></tr>
</table>

Date questionnaire completed __ / __ / __

Questions about adherence during assessment.

Keesense

1. Do you find the smart t-shirt _______ to use?
   - ☐ Very difficult
   - ☐ Fairly difficult
   - ☐ Neither difficult nor easy
   - ☐ Fairly easy
   - ☐ Very easy

2. How many days did it take you to fully master the smart t-shirt?
   I__I__I days

3. How often have you been wearing the t-shirt?
   - ☐ Day, how many hours I__I__I [hours]
   - ☐ Night, how many hours I__I__I [hours]
   - ☐ Day/Night, how many hours I__I__I [hours]

4. On average, over the last 3 days, how many hours per day did you you wear the T-shirt **for each of the following time slots** ?

**[0-6am]**	**[7-12am]**	**[1-6pm]**	**[7-12pm]**
☐ 1 hour	☐ 1 hour	☐ 1 hour	☐ 1 hour
☐ 2 hours	☐ 2 hours	☐ 2 hours	☐ 2 hours
☐ 3 hours	☐ 3 hours	☐ 3 hours	☐ 3 hours
☐ 4 hours	☐ 4 hours	☐ 4 hours	☐ 4 hours
☐ 5 hours	☐ 5 hours	☐ 5 hours	☐ 5 hours
☐ 6 hours	☐ 6 hours	☐ 6 hours	☐ 6 hours
☐ Not concerned	☐ Not applicable	☐ Not applicable	☐ Not applicable

5. On average, how many days per week did you wear the t-shirt?
   I__I__I days/week

6. According to you, the daily wearing of the t-shirt should be limited to I__I__I hours per day.

7. You were unable to wear ___________ your t-shirt due to a technical problem?
   - ☐ Often
   - ☐ Sometimes
   - ☐ Rarely
   - ☐ Never

Comments:

Date questionnaire completed __ / __ / __

Questions at the end of the evaluation

Elements	Questions
Satisfaction and Acceptability	
Perception, overall satisfaction	1. I am satisfied with the smart t-shirt.<br>1 ☐ 2 ☐ 3 ☐ 4 ☐ 5 ☐
	2. I felt comfortable with the use of the smart t-shirt.<br>1 ☐ 2 ☐ 3 ☐ 4 ☐ 5 ☐
Acceptability of the device (general comfort, use, activities)	3. I easily forget that I am wearing the smart t-shirt.<br>1 ☐ 2 ☐ 3 ☐ 4 ☐ 5 ☐
	4. I can accomplish my daily activities with the smart t-shirt.<br>1 ☐ 2 ☐ 3 ☐ 4 ☐ 5 ☐
	5. I can easily do physical activity with the smart t-shirt.<br>1 ☐ 2 ☐ 3 ☐ 4 ☐ 5 ☐
	6. I find the smart t-shirt easy to use.<br>1 ☐ 2 ☐ 3 ☐ 4 ☐ 5 ☐
Hygiene, physical or skin reaction	7. I sweat abnormally while wearing the smart t-shirt.<br>1 ☐ 2 ☐ 3 ☐ 4 ☐ 5 ☐<br>8. I have skin itching and/or irritation.<br>1 ☐ 2 ☐ 3 ☐ 4 ☐ 5 ☐
